# Trance states during a mind–body intervention monitored by BIS

**DOI:** 10.3389/fpsyg.2026.1728381

**Published:** 2026-01-30

**Authors:** Nina Zech, Martin Busch, Veronika Jaeger, Ernil Hansen

**Affiliations:** 1 Department of Anesthesiology, University Hospital Regensburg, Regensburg, Germany; 2 Private Practice, Dunningen, Germany

**Keywords:** Bispectral index, hypnosis, hypnotic susceptibility, mind–body intervention, trance

## Abstract

**Introduction:**

Mind–body interventions are increasingly used in health care, both for physical and for psychological therapy. With directing the focus to the body and inner experiences they include elements of trance induction and hypnosis. The scope of this experimental study was to test the hypothesis that a non-ordinary state of consciousness may occur during a mind–body intervention with changes in electrical brain activity similar to a hypnotic induction.

**Methods:**

Bispectral Index an EEG-derived parameter used in anesthesia, was monitored during a test of hypnotic susceptibility including a trance induction, and during a mind–body intervention modified from the Feldenkrais method. 54 adult volunteers were tested with a short version of the Harvard Group Scale of Hypnotic Susceptibility (HGSHS-5) and the instructions for a mind–body intervention from a 30-min audio file. The latter consists of an introduction, reflections on posture and sensations, and four suggested tasks with body movements and variations followed by a break each time. Contributing factors, namely age, sex, suggestibility and daytime, were analyzed by multifactorial analysis and linear regression.

**Results:**

BIS significantly decreased from an awake value of 97.5 to values of 88–92, similar in extent to the hypnosis induction of the test for HGSHS-5. Besides a general decline with duration significant drops were observed with every break. None of the tested potential influencing factors had significant impact.

**Conclusion:**

Measurable changes in electrophysical brain activity can be detected during a mind–body intervention as a surrogate marker of a state of non-ordinary consciousness. The high relevance arises from the fact that inclusion of the unconscious is essential for initiating beneficial changes in psychological and physical patterns, as these patterns are encoded and maintained there. The EEG reactions and aspects of a focus shift toward inside, body scan and confusion bring it close to hypnotic trance and provide new and objective research approaches.

## 1 Introduction

Recently, mind–body interactions have increasingly come into focus, both in psychological research and psychotherapy (Zhang et al., 2025). Neurological research has overcome the separation of body and mind also by neuroimaging techniques (Han et al., 2023). All mind–body interventions (MBI) focus on the interaction between the brain, body, and behavior and are practiced with intention to use the mind to alter physical function and promote overall health and well-being (Elkins et al., 2010; Wahbeh et al., 2008). Some of evolved mind–body trainings are rather considered techniques for Alternative and Complementary Medicine, while others are not only lifestyle- and wellness-directed but have scientific evidence für usefulness in medicine, e.g., movement disorders, rehabilitation after physical trauma (Berland et al., 2022; Phuphanich et al., 2020), neurology (Wahbeh et al., 2008), psychiatry (Martin et al., 2024), or pain therapy (Danon et al., 2022) and oncology (Deleemans et al., 2023; Elkins et al., 2010; Casuso-Holgado et al., 2022).

Personal experiences of similarities to hypnosis and of trance-like conditions during such MBI led us to study associated objective physiological changes like in EEG. In previous investigations we were able to demonstrate alterations in BIS during inductions of hypnosis (Zech et al., 2023). Bispectral Index (BIS) is a dimensionless numerical value derived from a patient’s electroencephalogram (EEG) that allows for easy and feasible monitoring of levels of consciousness especially during general anesthesia (Punjasawadwong et al., 2019) and sedation (Heavner et al., 2022).

The MBI we chose is not well known but shows high effectiveness on improvements for patients both of impaired muscular and psychic patterns and thus can stand for MBI in general. Based on principles of the Feldenkrais method (Buchanan and Ulrich, 2001), it incorporates body-scan elements (Gan et al., 2022) and uses body patterns as a metaphor for human functional pattern in general. In practical terms, it consists of selected body movements in rhythm with breathing, variations of the movements, reflections on posture and relationships between body regions, and breaks to allow transition of gained information for the brain from short- to long term memory (Busch, 2025). In contrast to most other mind–body therapies this approach does not use “exercises“, i.e., repetitions to come closer to a proposed optimal performance, but “experiments” with movements and reflections about how you do what you do that provides information to the brain to foster re-optimized functional patterns.

In this experimental study we tested the hypothesis that during a movement-based mind–body intervention instructed from a 30 min audio file EEG-changes are induced equivalent to a hypnotic trance induction. This is highly relevant because communication with, and involvement of the unconscious mind- an essential feature of hypnotic approaches- is crucial for facilitating favorable alterations in physical, physiological, and psychological patterns. They are anchored in unconscious processes and hardly reached by the critical conscious mind and deliberate decision-making (Landry et al., 2014; Hansen, 2024; Peter, 2024).

## 2 Materials and methods

### 2.1 Study design

For this prospective, experimental observational study, participants were recruited from the personal and professional environment of VJ, including students, members of a sports club, relatives, and physiotherapists. Inclusion criteria comprised an age between 18 and 76 years. Exclusion criteria included language barriers, impaired hearing, the inability to lie calmly and without pain on the floor, as well as major pre-existing medical conditions such as epilepsy, neurological or psychiatric disorders, or the current use of psychotropic medication.

A minimum sample size of 38 participants had been calculated based on power analyses from two previous studies conducted by the authors, which examined the effects of relaxation and hypnosis induction on Bispectral Index values. Of the 62 eligible individuals initially approached, 54 fulfilled the inclusion criteria and provided written informed consent following a screening interview. All measurements were conducted by individual appointment in a quiet, separate room to ensure standardized testing conditions.

### 2.2 BIS monitoring

During the study trial, an EEG-derived index was continuously recorded by a monitor for depth of anesthesia, namely the Bispectral Index Scale monitor (BIS-monitor, VISTA® monitoring system; Anandic Medical Systems, Switzerland). The BIS index is a numerically processed, clinically validated EEG parameter. Unlike traditional processed EEG parameters derived from spectral analysis, the BIS index is derived utilizing a composite of multiple advanced EEG signal processing techniques, including bispectral analysis, power spectral analysis, and time domain analysis. The key EEG features identified from the database analysis include the degree of beta or high frequency (14–30 Hz) activation, the amount of low-frequency synchronization, the presence of nearly suppressed periods within the EEG, and the presence of isoelectric periods within the EEG (Sigl and Chamoun, 1994). The exact algorithm for weighting and mathematical combination of these components to a single BIS value is unknown, since not publicly disclosed by the manufacturer for patent protection. Nevertheless, despite this scientific disadvantage BIS monitoring is widely used in clinical studies and practice. The BIS sensor is a single-use component consisting of a plaster with fixed electrodes. The sensor is placed with the central electrode at the center of the forehead, half a centimeter above the bridge of the nose, two electrodes above the left eyebrow, and an electrode midline between the edge of the eye and the hairline.

**Figure 1:**
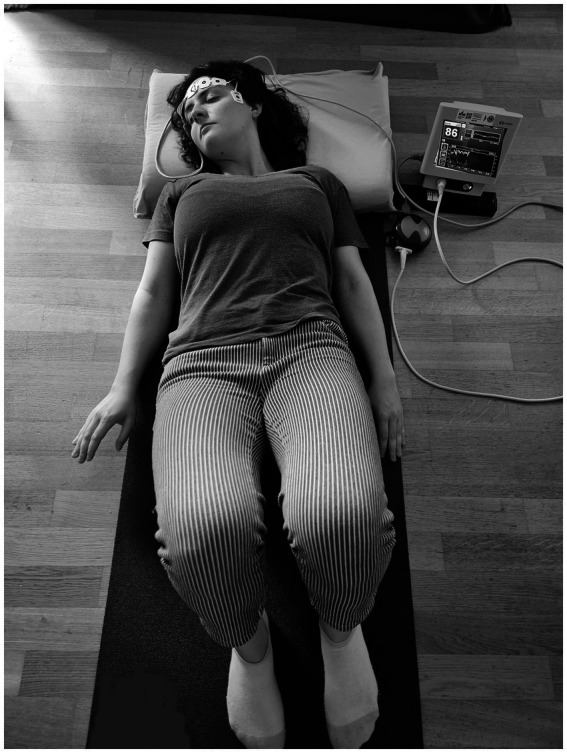
Measurement set-up. Study participant with BIS-monitoring during mind-body intervention. Figure contains images of the author(s) only.

The measurement was conducted in a quiet room to avoid any disturbance. For the test of suggestibility, participants were positioned on a comfortable chair with device-specific adhesive bilateral electrodes fixed on the forehead, following the manufacturer’s instructions. For the subsequent mind–body intervention, the test subject first sat on the floor on a comfortable mat with a pillow after the introduction the participant lay down on the mat to follow the instruction of the audio file. The test setting is shown in Figure 1.

For analyses, the first 90 s of the BIS-recording were discarded to correct for the latency time of the system. Baseline measurements of “Awake BIS” were carried out for several minutes each before and after HGSHS-5 testing and before and after MB intervention, respectively.

### 2.3 Suggestibility testing

**Figure 2:**
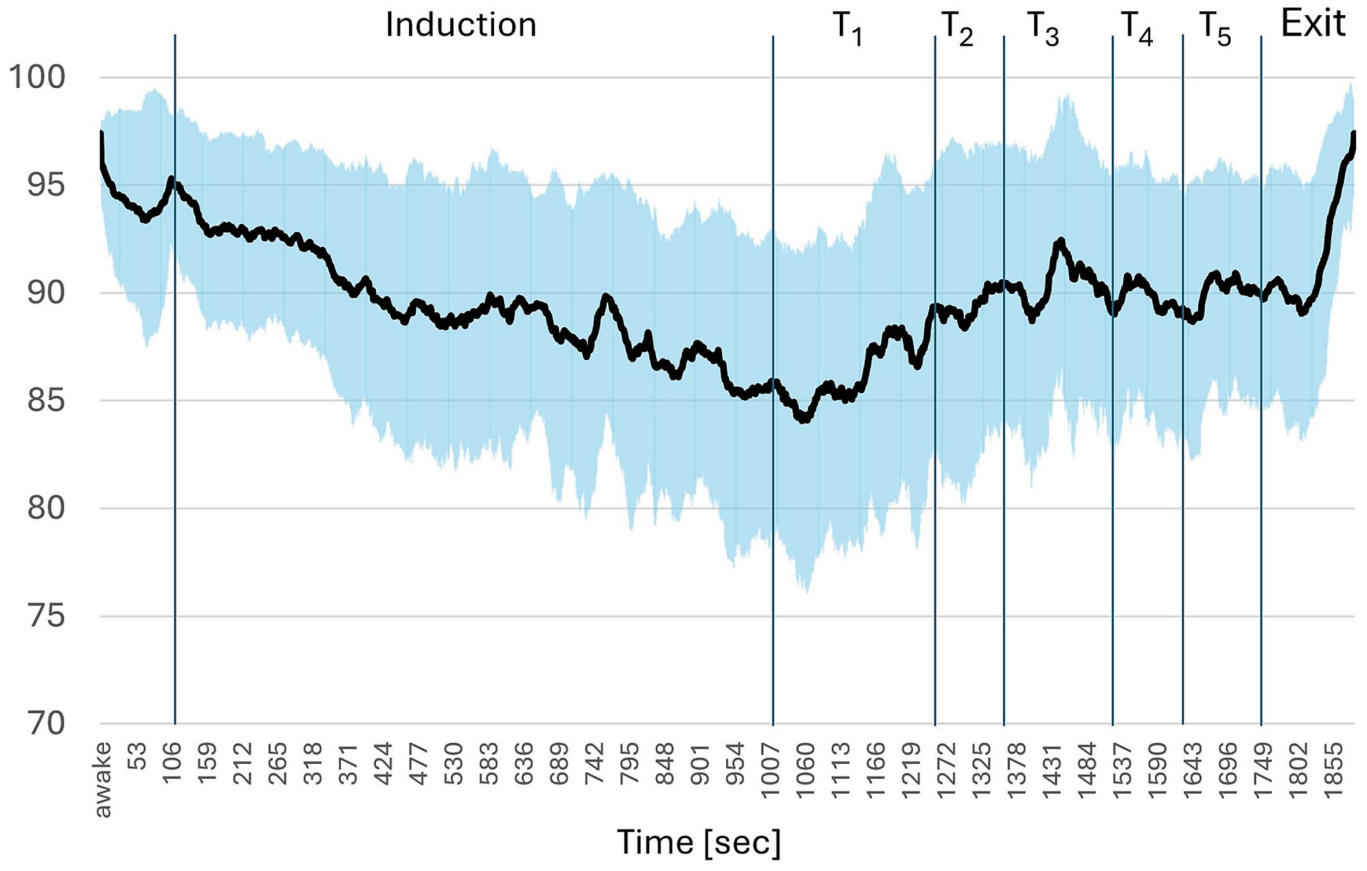
Bispectral index during suggestibility test (HGSHS-5). T = tasks 1–5 (*n* = 52).

After baseline measurements of BIS (several minutes “awake” value), every participant performed a test for hypnotic susceptibility. The HGSHS-5 (Riegel et al., 2021; Zech et al., 2024) is a shortened version of the HGSHS: A (Bongartz, 1985). The test takes approximately 31 min. An introduction (1.8 min) is followed by a hypnotic induction (15 min) resulting in eye closure. Five tasks follow that include the motor challenge items “arm immobility,” “finger lock,” “arm rigidity,” “head shaking inhibition,” and “eye catalepsy.” These tasks take another 12.4 min (see Figure 2). After the termination of the hypnotic trance, the efficacy of the suggestions, i.e., the quality with which each task was mastered, was evaluated by the subjects’ self-assessment by filling out the test questionnaire. Participants were rated according to the scores as “low suggestible” (low, scores 0–1), “medium suggestible” (medium, scores 2–3), and “high suggestible” (high, scores 4–5).

### 2.4 Mind–body intervention

The mind–body intervention according to Martin Busch consists of an introduction followed by a phase of reflections (break 0) and instructions for 4 tasks with subsequent breaks (Table 1). The text of the instructing audio file is given in the Supplementary material.

**Table 1:** Mind-body intervention with duration of the parts.

Part	Explanation	Time (min:sec)
Introduction		00:00
Break 0	Bodyscan	03:55
Task 1	Ellbow/Foot	05:30
Break 1		10:10
Task 2	Eyes/Head	11:45
Break 2		15:44
Task 3	Breathing	17:36
Break 3		22:44
Task 4	Eyes diagonal	24:00
Break 4		28:20
Exit	Eyes open, Reorientation	29:48
End		30:07

Starting out from the Moshe Feldenkrais’ approach that includes Awareness Through Movement and Functional Integration for the development of new patterns, the Martin Busch approach encourages experiments with variations of a movement instead of repeated practicing/exercising/training complex movement patterns. Directing attention through a movement suggestion that triggers different possible actions leads to the exploration of different variations (see also the “Introduction” in the text given as Supplementary material). To achieve this, language is precise in the description of “What” is done, and open to individual interpretation of “How” it is done. The instructions used here with the present audio file includes pressing one foot and the opposite elbow against the floor (task 1), movement of the eyes separately or together with the head from side to side (task 2), breathing with the hands uncrossed or crossed on upper or lower ribs (task 3), and eye movements together with the imagination of a diagonal between shoulder and opposite hip (task 4). The letter task includes the inexecutable suggestion of eyes moving forwards and backwards separately in the head.

Movements are performed in the rhythm of the breath, since breathing is also a movement (diaphragm, ribs), and controlled both consciously and unconsciously is a bridge to the unconsciousness. Moreover, this clock generator allows for individual and condition-adjusted pacing of the movements, in contrast to exercises, where breathing follows the movements. Even whether a movement goes with breathing out or in makes a difference. The patient is encouraged to observe “how you do what you do,” and again after variations of the movements. Thereby, the central nervous system receives new information, and the subsequent breaks give the brain the opportunity to compare the new information with its previous experiences, integrate new information and confirm what it already knows to optimize processes instead of practicing complex patterns.

For calculations, the phases of the MBI were subdivided to result in time periods of approximately equal length (1.5–2 min). The deepest phase regarding Bis was determined for every individual participant.

### 2.5 Statistical analyses

Variables were tested for normal distribution using the Kolmogorov–Smirnov-Lilliefors Test and visually. Accordingly, we present normally distributed variables with mean ± standard deviation (SD) and use a paired or unpaired Student’s t-test, as appropriate. Univariate ANOVA was performed including the 17 phases of the MBI. To assess potential contributing factors, multifactorial analyses of variance were conducted. The dependent variables were either “total intervention,” “all breaks,” “task 4b” (overall deepest phase), “deepest phase” (individual deepest phase), and “deepest minute.” As independent variables we used age group (15–30, 31–50, 51–80 years), gender, suggestibility group (low = 0–1, medium = 2–3, high = 4–5, according to the HGSHS-5) and daytime group. Linear regression analysis was performed with regard to the same dependent variables and the ungrouped independent variables: age, HGSHS-5 score and time of day. A *p* < 0.05 was considered statistically significant for all tests. All analyses were performed with IBM SPSS Statistics, Version 27.

## 3 Results

### 3.1 Baseline characteristics

Two test subjects rejected the suggestibility test (HGSHS-5 data missing). 20 of the 54 participants had previous experience with hypnosis and/or mind–body practices. The study population showed a left-skewed age distribution with three peaks indicated. Accordingly, three age groups were formed (Table 2). There were more female than male test subjects. The distribution of suggestibility scores according to HGSHS-5 was also left-biased and resulted in an unequal size of the three suggestibility groups.

**Table 2:** Baseline characteristics of patients.

Characteristics	Numbers
Total, *n* (%)	54 (100)
Age, years	
Median (interquartile range)	36 (22–53)
Mean (±SD)	38 (±16)
Age groups, *n* (%)	
15–30 years	21 (38.9)
31–50 years	19 (35.2)
51–80 years	14 (25.9)
Gender, *n* (%)	
Female	32 (59.3)
Male	22 (40.7)
Suggestibility groups	
Low (0–1)	39 (75%)
According to HGSHS-5 medium (2–3)	7 (13.5)
High (4–5)	6 (11.5)

### 3.2 BIS during suggestibility test

BIS decreased from an “Awake”-value of about 97.4 during trance induction to an average of 85 and increased to a level of around 90 in the course of the 5 tasks (Figure 2).

### 3.3 BIS during mind–body intervention

**Figure 3:**
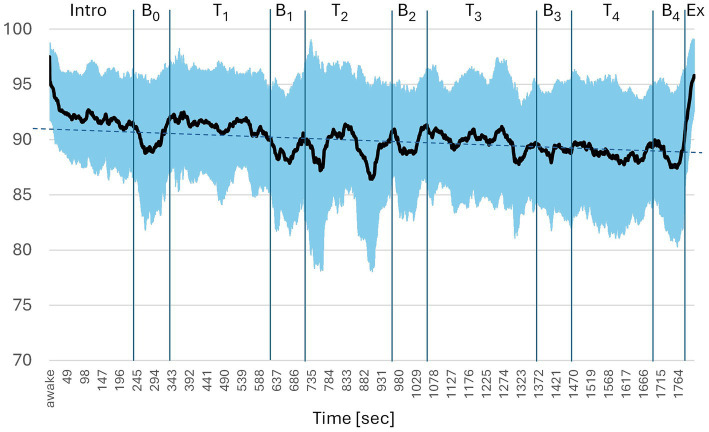
Bispectral index during mind–body intervention. Intro = introduction, B = breaks 0–4, T = tasks 1–4, Ex = exit; *n* = 54. Dotted blue line: linear regression of 1 min-intervals *y* = 91.43–0.11*x, R^2^ = 0.545, *p* < 0.001.

BIS decreased from an “Awake” value of about 97.5 during the introduction (lying down with eyes closed) to about 91 and, continuing in a fluctuating course with increasing lowering to about 88 (Figure 3). Noticeable drops occurred with every of the 5 breaks and to a varying degree during the 4 tasks. Overall, a continuous decrease of about one BIS-point per minute was observed in regression analysis, with a R^2^ of 0.545.

The average BIS during suggestibility test and mind–body intervention were not significantly different, while the difference between Awake-BIS and BIS during both interventions were highly significant (Table 3).

**Table 3:** Bispectral index during suggestibility test and mind–body intervention.

(*n* = 54)	BIS Awake	BIS total intervention	BIS deepest phase
Suggestibility Test (31.5 min, *n* = 52)	97.41 ± 0.26	89.62 ± 6.04	86.68 ± 6.55*
MBI (30.1 min, *n* = 54)	97.50 ± 0.23	90.10 ± 5.06	88.61 ± 7.51**
		*p* = 0.152	*p* = 0.603

The average BIS value during the total HGSHS-5-test and the total mind-body intervention was determined for each of 54 participants. Shown are the mean of 54 participants and the mean of the corresponding SDs. p was tested according to paired Student’s t-test. *: In HGSHS-5 the deepest phase (90 s after 17 min) is the end of the hypnotic induction. **: In the MBI the deepest phase is the second half of task 4 (T4b).

**Figure 4:**
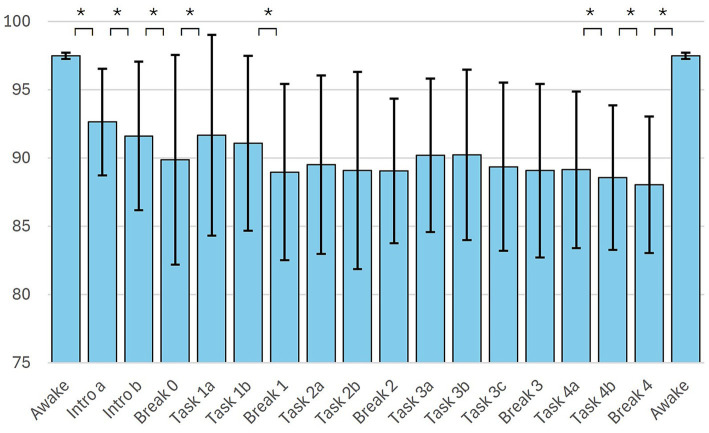
Phases of the mind-body intervention. Introduction and tasks were divided up so that they resulted in phases of approximately equal length (1.5–2 min); *n* = 54 * = *p* < 0.05.

The BIS during the different phases of the MBI is shown in Figure 4. For better comparison, the phases were subdivided to obtain roughly equal time periods of 1.5 to 2 min. The downward slope of BIS in the course of the intervention described above is also noticeable here. Significant drops in BIS were observed with almost every break.

### 3.4 Influencing factors

Multifactorial analyses showed no significant effect of the independent variables “age group” (*p* = 0.84–0.94), “sex” (*p* = 0.67–0.79), “suggestibility group” (*p* = 0.66–0.99) or “daytime group” (*p* = 0.68–0.89) on the target parameters (dependent variables) “total intervention,” “all breaks,” “task 4b” (overall deepest phase), “deepest phase” (individual deepest phase), and “deepest minute.” Linear regression analysis of the ungrouped independent variables “age” (R^2^ = 0.005–0.021), HGSHS-5 score (R^2^ = 0.001–0.030), and “time of day” (R^2^ = 0.029–0.054) with regard to those target parameters revealed no impact on the respective BIS-values.

Analysis of dependency of BIS-reduction on daytime showed a trend for HGSHS-5 (BIS total intervention) with 88.8 at noon vs. 90.5 in the morning and evening (*p* = 0.12). No significant differences were observed for the MBI (*p* = 0.69).

## 4 Discussion

### 4.1 Bispectral Index during mind–body intervention

The results of this study show that objectively measurable changes in the EEG occur during the mind–body intervention tested (see Figure 3). Throughout the intervention the BIS-values were significantly lower than in the awake state (see Table 3; Figure 4).

The extend of the BIS-reduction was comparable to that observed during hypnosis induction in the course of suggestibility testing (see Figure 2). It is noteworthy that the latter trance induction is not just a certain type of hypnosis induction but represents a standardized, worldwide used induction preceding the test for Harvard Group Scale of Hypnotic Susceptibility (HGSHS). Both interventions lead to a decrease in BIS to about 85. This corresponds to results of own preceding studies on HGSHS (Zech et al., 2023) or other types of hypnosis induction (publication in preparation) with BIS-monitoring. The variation within the test subjects was similar for BIS values during HGSHS-5-test and MBI (compare Figures 2, 3), namely about 6%. This also confirms previous results from BIS studies on trance (Zech et al., 2023).

While in the test for HGSHS-5, BIS is continuously decreasing during hypnotic induction and increasing during the subsequent tasks (see Figure 2), the course of BIS is varying during the MBI (see Figure 3). There are rapid and slow changes. Overall, the trend is declining over the 30-min experiment with a R^2^ of 0.545. This resembles the steady decline in BIS during the 15 min induction phase of HGSHS-5 testing and compatible with increasing trance depth.

Moreover, patterns of more rapid BIS changes can be identified, for instance a drop in BIS with every break. While during the exercises test participants are busy with movements, during the breaks they undertake reflections about their body position and relationships between different body parts. Such a shift in focus inward is known to be trance-inducing (Peter, 2024). In addition, the breaks are accompanied by relaxation and tranquility promoting a trance state. Drops in BIS possibly referring to trance deepening can be identified also during the exercises (see tasks 1–4 in Figure 3). Their origin remains unclear except for task 4, where in the second half participant are asked to move their eyeballs forwards and backwards. This represents an impossible task leading to confusion, which is a potent trance inducer (Erickson, 1964; Fusco et al., 2020).

The search for possible factors with impact on the observed effect of the mind–body intervention on BIS did not yield any significant results, for none of the selected BIS target parameters, namely “total intervention,” “overall deepest phase” (task 4b), “individual deepest phase,” “all breaks,” and “deepest minute.” Age, gender, hypnotic suggestibility (HGSHS), and time of day seem to have no influence on the observed changes in electrical brain activity. This is remarkable for the HGSHS, where a stronger reaction could be expected for “high suggestibles” (Bongartz, 1985; Peter, 2024). However, this is consistent with the low to missing impact of suggestibility grouping on hypnotic effects in clinical settings (Montgomery et al., 2011), and the lack of a correlation to BIS changes observed with hypnotic inductions (Zech et al., 2023). A dependency of hypnotic induction on the time of day with a higher suggestibility in the evening is generally assumed but not proven. An ultradian rhythm of susceptibility to trance and hypnotic phenomena have been described (Rossi, 1982) but is individually timed and thus escaped the present study. We also did not observe a higher susceptibility in females or a decrease with age as has been described for hypnotic susceptibility (Page and Green, 2007). These discrepancies indicate differences to hypnotic trance and phenomena despite the similarities in BIS.

### 4.2 Bispectral Index and trance

Changes in consciousness are based on brain processes which are reflected in electrical activity. The advantage of consideration of the EEG is the fast, almost online monitoring of brain activity, the non-invasive measurement, and its feasibility allowing continuous data collection in numerous persons. Extensive literature has examined the EEG correlates of hypnosis, reporting increased frontal alpha activity, decreases in central, frontal, and parietal gamma activity bilaterally, and increases in occipital gamma activity. The most consistently observed association between EEG dynamics and hypnosis has been found in the theta band (4–8 Hz). However, subsequent studies have not reliably replicated increases in alpha activity during hypnosis, and overall the findings remain inconsistent (De Pascalis, 2024). A bibliometric study on EEG research in hypnosis revealed limited validity of a “hypnosis fingerprint” because of methodological inconsistencies (Rinaldi et al., 2025). Other findings suggest “that hypnosis represents a modulation of the ordinary consciousness within its physiological variability rather than a distinct physiological state. Neither network nor topological differences account for the different subjective experiences of highs and lows” (Lucas et al., 2025). A review on neurophysiological characteristics of pain-directed hypnotherapy, comprising encephalography (EEG), magnetoencephalography, and evoked or event-related potentials summarize that research into hypnosis and hypnotically induced analgesia is primarily contradictory and has produced hardly any consistent results, although at a very rough level of analysis, the various studies showed on average clear differences in neuronal activation during the processing of pain under hypnosis and hypnotic analgesia compared to control conditions (Miltner et al., 2024). The situation with MBIs is similar. In a study on mindfulness meditation, for instance, EEG data revealed a significant decrease in traditional alpha (8–13 Hz) amplitude compared to rest, but no significant differences were observed between conditions in traditional delta, theta, beta, or gamma amplitudes (Duda et al., 2024). In any case, it is unlikely that a single mechanism can account for the full range of hypnotic phenomena. Hypnosis is a multifaceted process encompassing absorption, embodied relaxation, alterations of self-perception, and changes in the sense of agency; none of these components, in isolation, capture the entire spectrum of hypnosis phenomena. Neuropsychological evidence likewise confirmed the complexity of hypnotic phenomena and indicated that the neural mechanisms of hypnosis and hypnotizability are not fully understood (Terhune, 2015; Halsband and Wolf, 2019). Moreover, functional connectivity has come into focus of hypnosis research, rather than changes in EEG-frequencies and oscillatory patterns. Extending the question of EEG correlates to trance states further increases the complexity of observations.

The monitoring of the Bispectral Index as a processed EEG in contrast to sophisticated analysis of power spectra offers simple values ranging between 100 and 0 and give clinicians objective information about the depth of sedation. The BIS index is a numerically processed, clinically validated EEG-derived parameter. In contrast to traditional processed EEG measures that rely primarily on linear spectral analysis, the BIS algorithm incorporates multiple advanced signal-processing methods -including bispectral analysis, power spectral analysis, and time-domain analysis - to capture both linear and nonlinear features of cortical activity. In non-anesthetized individuals, modulations in BIS values predominantly reflect shifts along the upper range of cortical arousal. These shifts are typically driven by changes in attentional engagement, relaxation, internal focus, or reduced processing of external stimuli—factors directly relevant to the study of hypnotic induction and related states. The patient is considered awake for values over 90 (Jensen et al., 2004). General anesthesia comprises values ranging of 40 to 60, while deep sedation is within 60 to 70, and 70 to 90 represents light to moderate sedation. Although developed to monitor anesthetic depth, BIS is not strictly specific for anesthetics and sedatives but also affected by other chemicals like muscle relaxants or alcohol (Gerstman et al., 2015). It is also affected by physical (e.g., hypovolemia or, hypothermia) or metabolic (e.g., hypoglycemia) conditions (Dahaba, 2005). Moreover, changes in BIS do not exclusively reflect drug-induced effects on brain such as anesthesia and pharmacological sedation, but also non-pharmacological effects. Physiological sleep, for instance, has an effect on BIS and allows to distinguish the different sleep phases (Tung et al., 2002; Nieuwenhuijs et al., 2002). Moreover, applications of BIS monitoring outside the scope of the operating room and general ansethesia to “grade” other EEG conditions are considered (Dahaba, 2019). For instance, bispectral electroencephalogram monitoring has been used to assess neurologic status in unsedated, critically ill patients (Gilbert et al., 2001). Effects on BIS have also been demonstrated with methods of non-pharmacological relaxation and sedation, such as acupressure of the extra 1 acupoint (Fassoulaki et al., 2007) or mindfulness meditation (Turer et al., 2023).

Hypnotic trance is caused and accompanied by electrophysiological processes that can be observed in the EEG (Hinterberger et al., 2011; Wolf et al., 2022). Accordingly, also changes in BIS have been described (Dunham et al., 2021). In our previous study on BIS during the standardized hypnotic induction preceding suggestibility testing with the HGSHS, the electrophysiological measurements and the subjective score of hypnotic depth showed a similar course (Zech et al., 2023). The present experimental study supports the hypothesis that during a movement-based mind–body intervention EEG-changes are induced similar to a hypnotic trance induction. This demonstration of trance states during MBI is of central importance, as body functions, behavior and psychological performance are all predominantly determined unconsciously, and can only be accessed and modified there (Landry et al., 2014; Peter, 2024). Although hypnosis represents a particularly suitable access to the unconscious, also MBI involves the unconscious and can trigger modifications in physiological and psychological patterns. Thus, using electrophysiological parameters not strictly specific for hypnosis could be advantageous.

### 4.3 Study limitations and outlook

The BIS value is a composite parameter of various EEG features, and the algorithm for combining and weighting of these variables is not disclosed by the monitor manufacturer. This means a considerable limitation in terms of significance and scientific research. However, the aim of the presented study was not a comparison of EEG parameters and wave ranges, but the demonstration of intervention-induced changes in consciousness.

No objective measure is available to date for trance and trance depth. The EEG changes monitored by BIS used here can only be seen as a surrogate marker. Nevertheless, the utilization of a method originally developed to monitor anesthetic depth is not so unreasonable because intravenous drugs inducing general anesthesia are named “hypnotics.” Moreover, BIS changes caused by physical conditions, by drugs or by sleep can be excluded for the present study. It is therefore quite likely that an equivalent of non-pharmacological sedation or trance is being depicted by the reported results. And even without the possibility for a clear correlation with a hypnotic or natural trance state, the described easy and feasible measurement could help to study further and to reinforce trancelike aspects of mind–body interventions.

No clear assignment of the measured electrophysiological effects to hypnotic trance or other trance states is possible. The resting position during the measurement and some elements of the mind–body intervention such as body scan could indicate a connection to other psychological aspects like deep relaxation or meditation. Such phenomena including trance can be summarized as “non-ordinary states of consciousness,” and recent neurophysiological research finds similarities and general functional characteristics beside specific features (Kumar et al., 2024; Moujaes et al., 2024). Therefore, an exact and unequivocal designation of the study’s correlate and commitment to “hypnotic trance” despite demonstrated similarities might not be necessary and even helpful. Leaving the everyday state of consciousness during mind–body interventions is an aspect deserving further consideration and research.

The demonstration of a comparable reduction in ordinary awake consciousness, with BIS as a surrogate marker, is compatible with the hypothesis that this is a common essential of MBIs and hypnosis to reach and modulate programmed patterns in the unconscious.

## Data Availability

The raw data supporting the conclusions of this article will be made available by the authors, without undue reservation.
